# ALDH^HIGH^ Population Is Regulated by the AKT/β-Catenin Pathway in a Cervical Cancer Model

**DOI:** 10.3389/fonc.2020.01039

**Published:** 2020-07-17

**Authors:** Miguel Ángel Sarabia-Sánchez, Eduardo Alvarado-Ortiz, Mariel Esperanza Toledo-Guzman, Alejandro García-Carrancá, Elizabeth Ortiz-Sánchez

**Affiliations:** ^1^Programa de posgrado en Ciencias Bioquímicas, Facultad de Química, Universidad Nacional Autónoma de Mexico, Mexico City, Mexico; ^2^Unidad de Investigación Biomédica en Cáncer, Instituto de Investigaciones Biomédicas, Universidad Nacional Autónoma de México and Instituto Nacional de Cancerología, Secretaría de Salud, Mexico City, Mexico; ^3^Subdirección de Investigación Básica, Instituto Nacional de Cancerología, Secretaría de Salud, Mexico City, Mexico; ^4^Programa de posgrado en Ciencias Biológicas, Facultad de Ciencias, Universidad Nacional Autónoma de Mexico, Mexico City, Mexico

**Keywords:** β-catenin, GSK3-β, ALDH, cervical cancer, AKT

## Abstract

ALDH is an enzyme involved in different cellular processes, including cancer. It has been shown that a cellular subpopulation with high ALDH activity (ALDH^HIGH^) within a tumor is related to functional capabilities such as stemness, chemoresistance, and tumorigenicity. However, few studies have focused on determining the mechanisms behind ALDH activity within the cells. Previously, our group reported that ALDH^HIGH^ cells have higher tumorigenicity in Cervical Cancer (CC) cell lines. Based on this, we were interested to know the molecular mediators of the ALDH^HIGH^ cells, specifically β-catenin, inasmuch as β-catenin is regulated through different pathways, such as Wnt signaling, and that it acts as a transcriptional co-activator involved in cancer progression. In this work, we show that the increase in ALDH^HIGH^ cell percentage is reverted by β-catenin knockdown. Consistently, upon GSK3-β inactivation, a negative regulator of β-catenin, we observed an increase in ALDH^HIGH^ cells. Additionally, we observed a low percentage of cells positive for Fzd receptor, suggesting that in our model there is a low capacity to respond to Wnt ligands. The analysis of ALDH^HIGH^ cells in a sphere formation model demonstrated the active state of AKT. In accordance with this, impairment of AKT activity not only reduced β-catenin active state, but also the percentage of ALDH^HIGH^ cells. This corroborates that AKT acts upstream of β-catenin, thus affecting the percentage of ALDH^HIGH^ cells. In conclusion, our results show that ALDH^HIGH^ cells are dependent on β-catenin, in spite of the Wnt pathway seems to be dispensable, while AKT emerges as central player supporting a mechanism in this important axis that is not yet well known but its analysis improves our understanding of ALDH activity on CC.

## Introduction

The Aldehyde dehydrogenase (ALDH) enzyme catalyzes the conversion of endogenous and exogenous aldehydes to carboxylic acids, being a key factor for improving the detoxification and chemoresistance associated with cancer relapse. Moreover, it has been shown that an increase in ALDH protein levels and/or ALDH-related enzymatic activity is associated with poor clinical prognosis in different types of cancer (1, 2). Encompassed by a family of isoenzymes, ALDH proteins have multiple functions in cancer cells, including proliferation, differentiation, and pharmacological resistance.

There is also evidence that high ALDH activity (ALDH^HIGH^) improves stemness-related characteristics reflected on Cancer Stem Cells (CSC). CSCs are a cellular subpopulation able to self-renew and differentiate into the rest of the cells in the tumor mass. Furthermore, employing spheres formation cultures, it has been possible to enrich CSC-associated characteristics identified not only by ALDH^HIGH^ activity, but also through other molecular markers associated with CSC populations, for instance CD44, CD24, LGR5/GPR49, among others. Particularly in Cervical Cancer (CC), spheres cultures from cell lines display higher levels of CSC-related molecular markers as compared with monolayer cultures, such as Oct-4, Nanog, and CD49f. Moreover, studies from our and other groups recognized that ALDH^HIGH^ cells were enriched in spheres cultures and are associated with tumorigenic capacities and resistance to cisplatin (3–5).

In the last years, the understanding of mechanisms that regulate the levels and activity of ALDH has received special interest. However, molecular signaling explaining this enzymatic activity are not well elucidated. Thus, we were interested in β-catenin, a crucial transductor previously associated with CSC-related markers (6). It is well known that β-catenin is an important component of canonical Wnt pathway and regulates cellular processes such as adherent junctions (7). In this sense, upon absence of Wnt ligands, β-catenin can be a target of sequential phosphorylations at its N-terminal region, by CKI-α and GSK3-β proteins, resulting in its proteosomal degradation promoted through β-TrcP (8–10). In presence of Wnt ligands, the protein complex that normally drives β-catenin degradation is uncoupled, and therefore β-catenin can translocate to the nucleus acting as transcriptional coactivator for the family of transcription factors TCF/LEF, leading to the expression of target genes involved in proliferation, having positive repercussions on tumorigenic cell capabilities. Currently, it is well known that aberrant cellular signaling of β-catenin is a characteristic feature of cancer progression, because abnormal intracellular distribution of β-catenin has been observed in cancerous samples compared to normal tissues (9, 11, 12). Interestingly, nuclear localization of β-catenin seems to favor initiation and progression of different types of cancer, and CC is not the exception (13, 14).

Recently, it has been reported that TCF/LEF transcription factors are able to bind to the ALDH promoter, acting as positive regulators of this gene (15). Nevertheless, the mechanisms explaining the activation of β-catenin are not totally understood in cancer and, moreover, such activation could depend on multiple contexts and extracellular signals (5). Therefore, the aim of this work was to study the activity of β-catenin on ALDH, as well as to evaluate the regulators that carry out this activation in case of CC. We reveal that ALDH is positively regulated by β-catenin, but interestingly, this effect is downstream of AKT activity and is mediated by a FZD-independent mechanism. This supposes an important axis for the establishment of a malignant behavior in cervical cells.

## Materials and Methods

### Cell Culture

The cervical cancer cell line SiHa (HPV16 squamous cell carcinoma) was purchased from ATCC (American Type Culture Collection, Manassas, VA, USA). SiHa cells were cultured in Dulbecco's Modified Eagle's Medium (DMEM) (Gibco®), and the medium was supplemented with 10% Fetal Bovine Serum (FBS) (Gibco®). The cells were incubated in a humidified atmosphere to 37°C containing 5% CO_2_. SiHa cells were used for spheres cultures cell culture in MammoCult™ medium (Stem Cell Technologies®) complemented with heparin, and antibiotic according to the concentrations suggested by the supplier. The density of cell culture was 9,000 cells/mL, using 8 mL in 100 mm ultra-low attachment culture dish (Corning®). The spheres cultures were cultured for 72 h.

### Pharmacological Treatment

For pharmacological assays, 2 × 10^5^ cells were seeded in 35 mm culture dishes, and incubated overnight. Cells were treated with Bromo-Indirubin-3'-Oxime (BIO) (Cat. No. 667463-62-9, Sigma Aldrich®) or control (Vehicle DMSO) at specified concentrations during 48 h. After incubation, cells were harvested for Flow Cytometry and Western Blot assays. Inhibition of AKT activity was performed in spheres cultures over 72 h using AZD-5363 at specified concentrations, DMSO was used as negative control.

### β-Catenin Knockdown Assays

β-catenin was targeted using SMARTpool siGENOME platform. All transfections were done using Lipofectamine 3000 (Invitrogen®) in Opti-MEM (Gibco®) according to protocol conditions. 100 nM of SMARTpool siGENOME *CTNNB1* siRNA and siGENOME Non-Targeting siRNA #1 (Dharmacon®), referred hereinafter as siRNA β-catenin and Scramble, respectively, were used for β-catenin knockdown. After 24 h, the medium was removed, and posterior assays were carried out.

### β-Catenin Transcriptional Activity Assays

To evaluate TCF/LEF transcription activity, the pTOP/FOP plasmid system was used as described previously (15). Luciferase activity was performed after co-transfection of pTOPFlash or pFOPFlash and β-Galactosidase (40 and 10 ng, respectively) as internal control in 24-Well Plates. After 24 h, the pharmacological assay was carried out and the activity was evaluated using luciferase reporter system (Promega®) based on protocol specifications. Additionally, Tropix Galacton Plus (Thermo Scientific®) was employed to evaluate β-Galactosidase in the same tube.

### Western Blotting

The cells were lysed using lysis buffer (50 mM Tris Base, 5 mM EDTA, 133 mM NaCl, 1 mM PMSF, 1% Triton X-100, and 1X Phosphatase Inhibitor Cocktail) and were preserved to −70°C until their use. Then, protein extracts were quantified using the Pierce™ BCA Protein Assay kit (Thermo Scientific®). The proteins were separated using equal concentrations for each experimental condition using 10–12% Sodium Dodecyl Sulfate-Polyacrylamide Gel Electrophoresis (SDS-PAGE). Proteins were transferred to a nitrocellulose membrane and then, the membrane was blocked using 5% milk free-fat in TBS at room temperature for an hour. Primary antibodies were incubated overnight as follows: Non-phospho (Active) β-catenin (Cat. No. 8814, Cell Signaling®) 1:2000, β-catenin (14-2567-82 eBioscience®) 1:3000, p-AKT1/2/3 ser-473 (sc-7965, Santa Cruz Biotechnology®) 1:200, total AKT (sc-1618, Santa Cruz Biotechnology®) 1:1000, p-GSK-3β (sc-373800, Santa Cruz Biotechnology®) 1:200, Lamin A/C (sc-6215, Santa Cruz Biotechnology®) 1:1000, ALDH (Cat. No. 611195, BD Transduction Laboratories®) 1:200, and GAPDH (sc-48167, Santa Cruz Biotechnology®) 1:5000 dilution. Secondary antibody was incubated for 1 hour based on following conditions: anti-Goat IgG-HRP (sc-2320, Santa Cruz Biotechnology®) 1:10000, anti-Rabbit IgG-HRP (sc-2013, Santa Cruz Biotechnology®) 1:5000, and anti-Mouse IgG-HRP (sc-2005, Santa Cruz Biotechnology®) 1:5000. Blots were revealed using Immobilon™ Western chemiluminescent HRP substrate (Millipore®, WBKLS0500) and employing C-DiGit (Li-Cor).

### Cellular Fractionation

Cellular fractionation was performed according to protocol of the NE-PER^TM^ Nuclear and Cytoplasmic Extraction Reagents kit (Cat. No. 78835, Thermo Scientific®). Cell cultures were harvested, washed with PBS 1 × and 1 × 10^6^ cells were resuspended into CER I buffer. Cells were shaken vigorously and then CER II buffer was added. Cells were centrifuged at 14,000 rpm for 5 min. The supernatant was recovered and stored as cytoplasmic extract. The pellet was resuspended in NER buffer and shaken vigorously every 10 min for 40 min. Finally, cells were centrifuged at 14,000 rpm for 10 min. The supernatant was recovered and stored as nuclear extract.

### Immunofluorescence Staining

Cells were seeded on slides and cultured until reaching 80–90% confluence. Cells were fixed in 4% paraformaldehyde for 5 min. Then, cells were permeabilized with 0.3% Triton X-100 for 1 h and blocked with 10% FBS. Cells were then incubated with anti-human/mouse β-catenin (Cat. No. 14-2567-82, eBioscience®) 1:200 overnight at 4°C. Following incubation, cells were washed with PBS 1X and incubated with fluorescein horse anti-mouse IgG secondary antibody (Cat. No. FI-2000, Vector Laboratories Inc®) for 2 h at room temperature. Finally, cells were washed with 1X PBS and nuclei were stained with propidium iodide for 5 min at room temperature. The images were captured using Confocal Microscope Nikon A1R+ STORM.

### Flow Cytometry

Fzd receptor was detected as follows, cells were harvested and washed with 1X PBS, then the cells were incubated in absence or presence of primary anti Frizzled antibody (sc-9169, Santa Cruz Biotechnology®) at 1:100 dilution in Flow Buffer (0.5% BSA, 2 mM EDTA, PBS1X) for 30 min at 4°C. After incubation, both conditions were centrifuged for 5 min and the supernatant was removed. Then, the cells were incubated with fluorescein Goat anti-rabbit IgG secondary antibody (Cat. No. Fl-1000, Vector Laboratories®) diluted in Flow Buffer, for 30 min at 4°C protected from the light. Cells were then centrifuged for 5 min and the supernatant was removed. The cells were resuspended in Flow Buffer and acquired as described below. Cells incubated in absence of primary antibody were considered as negative control.

ALDH activity was evaluated according to the ALDEFLUOR^TM^ kit (Stem Cells Technologies®) protocol. After treatments, cells were harvested and washed with 1X PBS. Then, cells were resuspended in 1 mL of ALDEFLUOR^TM^ assay buffer. For the test tube, 0.609 μg of ALDEFLUOR reagent were added to 1 × 10^6^ cells resuspended in 1 mL of ALDEFLUOR^TM^ assay buffer. As negative control, cells were treated with 5 μM of Diethylaminobenzaldehyde (DEAB), a specific ALDH inhibitor that is used for background fluorescence. Test and control tubes were incubated for 45 min at 37°C protected from the light. After incubation, both tubes were centrifuged for 5 min and the supernatant was removed. The cells were resuspended in ALDEFLUOR^TM^ assay buffer. Cells were acquired in a BD FACSCalibur cytometer and 50,000 events were recorded. Based on control tube (DEAB), cells were sorted as ALDH^LOW^ and ALDH^HIGH^ cells using FACSJazz™ Becton Dickinson sorter. Data analysis was performed using the FlowJo Software (Tree Star, USA).

### GEPIA Analysis

Gene Expression Profiling Interactive Analysis (GEPIA) was used to determine the overall survival and the correlation analysis in gene expression of patients with Cervical Squamous Cell Carcinoma and Endocervical Adenocarcinoma (CESC) obtained of Cancer Genome Atlas (TCGA) and Genotype-Tissue Expression (GTEx). In the case of overall survival, the system used the Mantel-Cox test with quartile cutoff (75% high and 25% low), while spearman coefficient was used for correlation analysis (16).

### Statistical Analysis

Data are represented as means ± Standard Error of the Means (S.E.M.). The analysis was done using ANOVA one-way followed by Dunnet *post hoc*, while Student's t-test was employed to compare means of two groups. *p* < 0.05 was considered as statistically significant.

## Results

### β-Catenin Is Involved in the Enrichment of ALDH^HIGH^ Cells in Spheres

**Figure 1:**
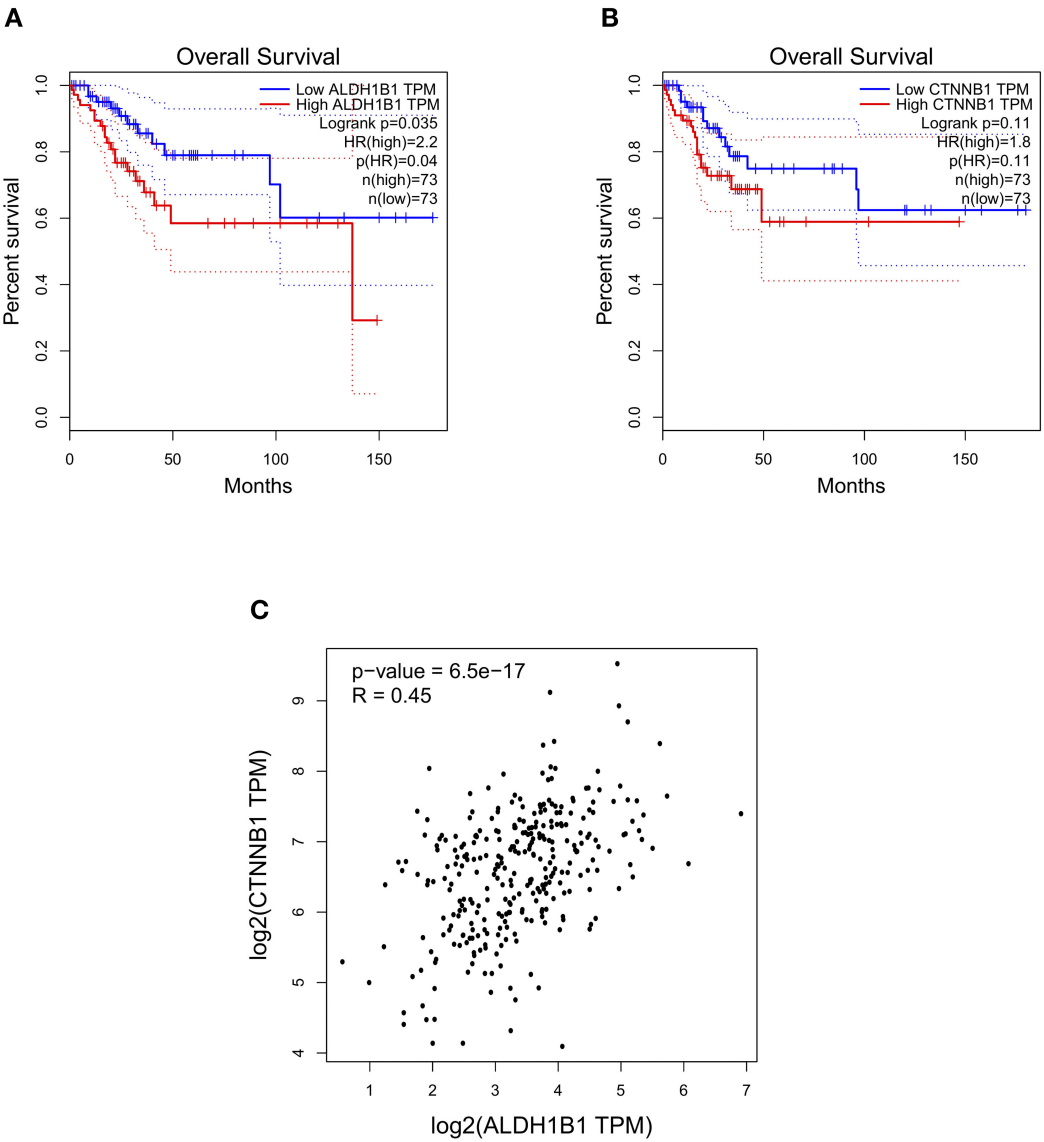
*ALDH1B1* and *CTNNB1* gene expression show positive correlation in cervical cancer patients. **(A,B)** Overall survival curves obtained from cervical squamous cell carcinoma and endocervical adenocarcinoma patients. **(C)** Pair-wise correlation between *ALDH1B1* and *CTNNB1* using the Spearman method. Graphs were obtained from GEPIA database.

The ALDH enzyme family is encoded by 19 ALDH functional genes. Hence, in order to determine the members of ALDH family that could have prognostic relevance in CC, we used the GEPIA data base to determine the Overall Survival (OS) of patients with Cervical Squamous Cell Carcinoma (CSCC) and Endocervical Adenocarcinoma (EA) based on levels of gene expression from each ALDH gene. The results showed that high levels of *ALDH1B1* transcript were significantly associated with poor OS in patients with CSCC and EA (Figure 1A). To determine whether β-catenin, encoded by *CTNNB1* gene, has a comparable relationship to ALDH1B1 on same type of cancer, a pair-wise gene expression determination was carried out. As shown in Figure 1B, high levels of *CTNNB1* transcript had a similar association with poor OS of patients with CSCC and EA. Furthermore, *ALDH1B1* and *CTNNB1* have a positive correlation in the same samples of patients with CC (Figure 1C), suggesting that both could participate in the same molecular mechanism, and explaining the similar relevance in clinical data.

**Figure 2:**
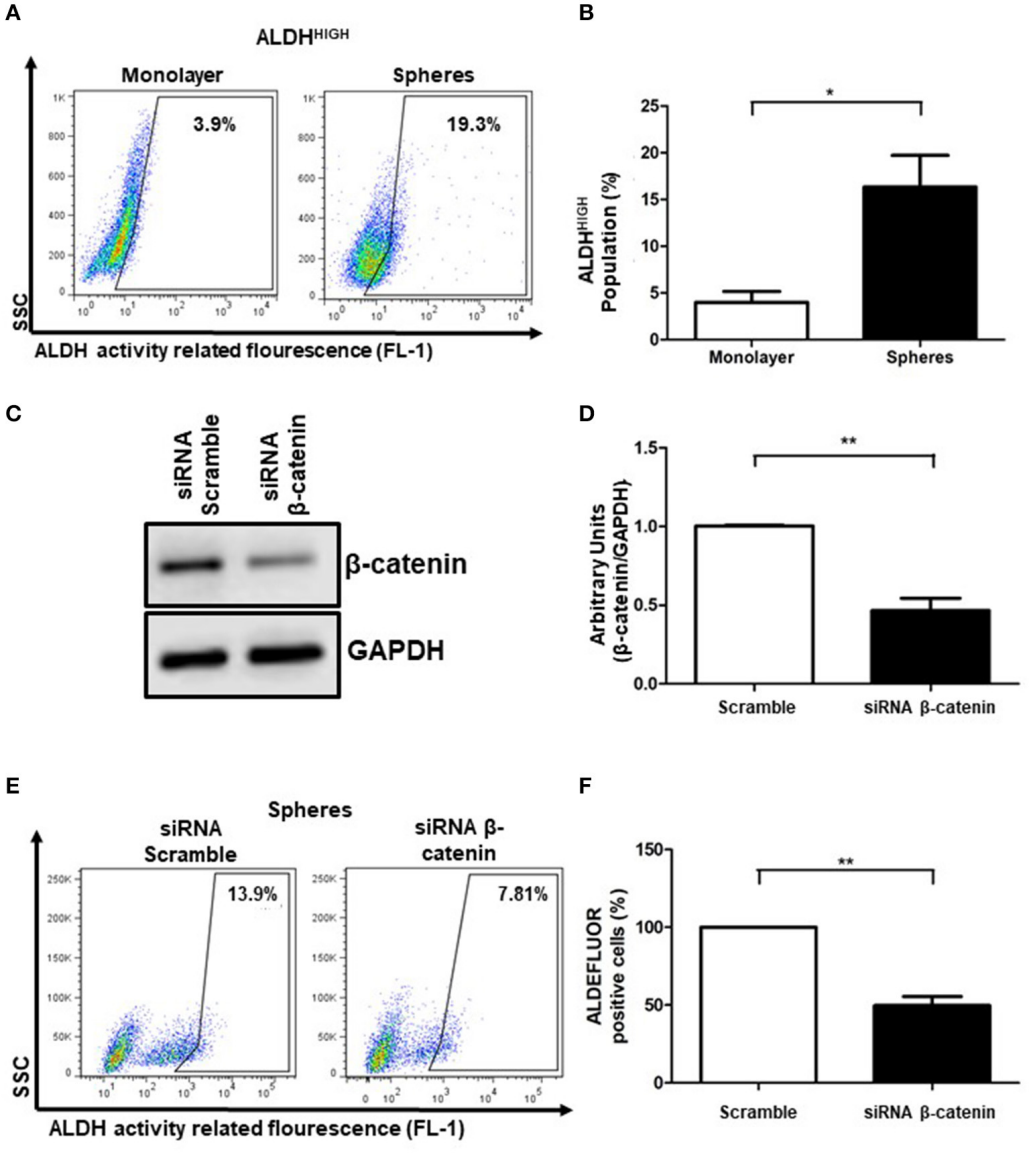
β-catenin is related to ALDH^HIGH^ activity cell population**. (A,B)** Density plots showing the percentage of ALDH^HIGH^ cells in SiHa cell line grown in monolayer or spheres cultures. The bar graph showed the percentages of ALDH^HIGH^ cells in monolayer (white bar) or spheres cultures (black bar). The enzymatic activity of ALDH was measured by flow cytometry using the ALDEFLUOR kit. The inhibitor DEAB was employed as a background fluorescence control (data not shown). Data was normalized with DEAB (internal control) from 3 independent experiments are showed in bar graph (Means ± S.E.M.), *p < 0.05. **(C)** Western Blot of β-catenin in SiHa cells transfected with siRNA scramble (negative control) or siRNA β-catenin at 100 nM during 24 h. GAPDH was used as loading control. **(D)** The units were normalized with siRNA scramble and the densitometry quantification from 3 independent experiments are showed in bar graph (Means ± S.E.M), **p < 0.01. **(E,F)** Density plots showing the percentage of ALDH^HIGH^ cells obtained of spheres cultures from SiHa cells transfected with siRNA scramble (left panel) or siRNA β-catenin (right panel). Transient transfection of SiHa cells using siRNA scramble (control) or siRNA β-catenin was employed to knockdown β-catenin. At 24 h post-transfection, SiHa cells were growth in spheres cultures for an additional 72 h. Each percentage of ALDH^HIGH^ cells was normalized with DEAB as internal control. The percentages of ALDH^HIGH^ cells with siRNA β-catenin were normalized to percentages of ALDH^HIGH^ cells with siRNA scramble. The data from at least 3 independent experiments are shown in bar graph (Means ± S.E.M.), **p < 0.01.

We used sphere formation culture as a method to enrich the ALDH^HIGH^ subpopulation, given that monolayer adherent cultures show low percentage of ALDH^HIGH^ cells. Employing these tridimensional cell cultures, it has been possible to improve characteristics of stemness, pharmacological resistance, tumorigenic capabilities, among others (1, 4). To evaluate ALDH^HIGH^ percentages, we used the ALDEFLUOR assay that allows us to determinate the percentages of ALDH^HIGH^ cells based on the enzymatic activity of different members of the ALDH family. As expected, the percentage of ALDH^HIGH^ cells was lower under standard monolayer conditions, however this percentage was enriched in the cells that formed spheres (Figures 2A,B). Consistently, the cellular distribution of active β-catenin in spheres showed higher percentages in nuclear fraction compared to cytoplasm (Supplementary Figure 1). These results showed that the spheres cultures represents a tool that allows us to have a greater percentage of ALDH^HIGH^ cells. We used the inhibitor DEAB as internal control in each condition to determine the percentage of ALDH^HIGH^ cells (data not shown).

Since members of ALDH family, such as ALDH1A1 gene, can be transcriptionally regulated by β-catenin (17), we evaluated the relevance of β-catenin in the ALDH^HIGH^ cell enrichment phenomena in SiHa spheres. Interestingly, after β-catenin knockdown (53.71%) (Figures 2C,D), the percentage of ALDH^HIGH^ cells were also reduced (Figures 2E,F).

**Figure 3:**
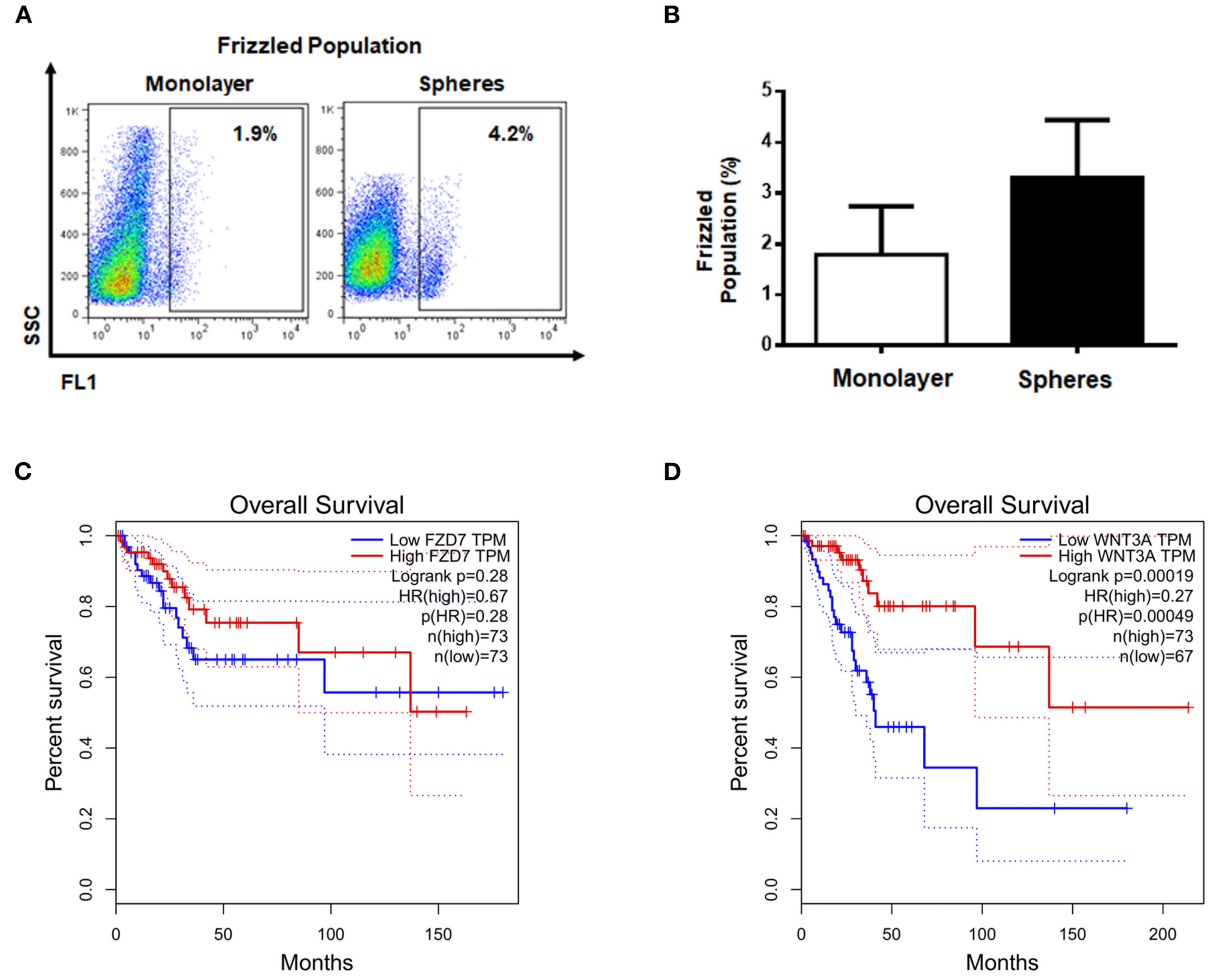
The survival of cervical cancer patients is not related to elements of canonical Wnt pathway. **(A,B)** Representative diagram of Fzd^+^, the values shown the percentages of positive cells from monolayer and spheres cultures, secondary antibody was used as autofluorescence control (not shown). The bar graph shown the percentages data from at least 3 independent experiments. **(C,D)** Survival patients curves of *FZD7* and *WNT3A* obtained from GEPIA data base.

It is known that β-catenin can be regulated by different extracellular factors such as Wnt ligands, which are recognized by Fzd receptors. Therefore we were interested in knowing whether the percentages of Fzd^+^ cells were comparable to the percentage of ALDH^HIGH^ cells, which could indicate a relationship between ALDH and the Wnt pathway. For this reason, we used an antibody able to recognize all members of Fzd family. The flow cytometry analysis showed that both monolayer and spheres cultures had a low percentage of Fzd^+^ cells (Figures 3A,B). However, in both cases the means of percentages of Fzd^+^ cells were even lower than ALDH^HIGH^ cells, which suggested that the two subpopulations are not necessarily related.

To discern if, despite of low percentage of Fzd^+^ cells, Fzd has relevance in CC samples, the OS of patients with CSCC and EA was determined for Fzd7, a prototypical receptor of Wnt signals. The results showed that *FZD7* transcript was not significantly correlated with the OS of patients with CSCC and EA (Figure 3C). Additionally, we analyzed *WNT3A* transcript levels, the most studied ligand that is able to activate the β-catenin-mediated pathway, employing GEPIA data base of the same CC samples. Interestingly, *WNT3A* exhibited an opposite correlation to that observed for *ALDH1B1* and *CTNNB1* transcripts, meaning that low levels of *WNT3A* transcript were associated with poor OS of patients with CSCC and EA (Figure 3D). These results implied that Wnt3a and Fzd might be dispensable in the molecular association between *ALDH1B1* and *CTNNB1* transcripts.

### Inhibition of GSK3 Increases ALDH^HIGH^ Percentage Cells in a β-Catenin-Dependent Manner

**Figure 4:**
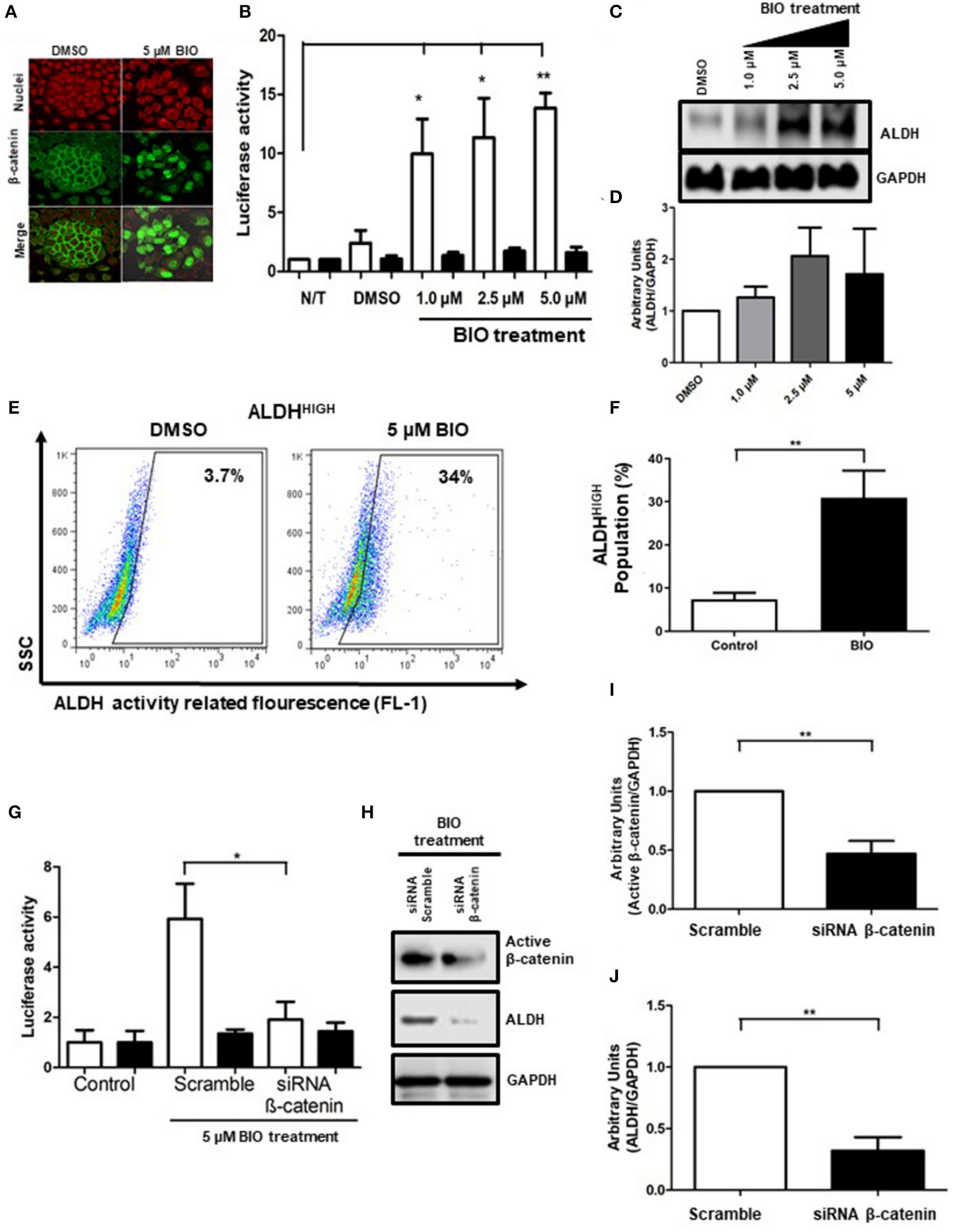
GSK3-β inhibition upregulates ALDH through β-catenin-dependent transcriptional activity. **(A)** Images showing β-catenin (green) localization in SiHa cells treated with DMSO (vehicle) or 5 μM of BIO obtained by immunofluorescence microscopy. The nuclei were stained with IP (red). Representative images from at least 3 independent experiments are showed. Magnification 40X. **(B)** Reporter activity of β-catenin-dependent transcription in SiHa cells exposed to BIO treatment. Transient transfection of SiHa cells using the plasmid control pFOP (black bars) and the reporter plasmid pTOP (white bars) was employed to measure transcriptional activity of β-catenin. At 24 h post-transfection, the SiHa cells were treated with BIO at the concentrations indicated for an additional 48 h. The values of luciferase were normalized to β-galactosidase and the data are showed in bar graph from 5 independent experiments, (Means ± S.E.M). *p < 0.05, **p < 0.01. **(C,D)** Western Blot of ALDH in SiHa cells growth as monolayer culture and exposed to BIO at the concentrations indicated during 48 h. GAPDH was used as loading control. Densitometry quantification is showed in bar graph (Means ± S.E.M). **(E,F)** Density plots showing the percentage of ALDH^HIGH^ cells in SiHa cells treated with DMSO (vehicle) or BIO. The enzymatic activity of ALDH was measured by flow cytometry using the ALDEFLUOR kit. DEAB inhibitor was employed to determine the percentage of ALDH^HIGH^ cells for each condition (data not shown). The percentages were normalized with DEAB (internal control) and the data from 3 independent experiments are showed in bar graph (Means ± S.E.M.), *p < 0.05, **p < 0.01. **(G)** The activity of TCF/LEF promoter from transfected cells with 100 nM siRNA scramble or siRNA β-catenin and stimulated with BIO treatment for 48 h (Means ± S.E.M), *p < 0.05. **(H-J)** Western Blot analysis of active β-catenin and ALDH after BIO treatment for 48 h, densitometry quantification of 3 independent experiments is showed in bar graphs (Means ± S.E.M), **p < 0.01.

Since Fzd receptors are not associated with ALDH^HIGH^ cells, and since β-catenin is important, we evaluate GSK3, a direct regulator of β-catenin, which acts as an antagonist by promoting β-catenin degradation through phosphorylation. Over the past few years, different inhibitors targeting GSK3 have been developed (18). In the present work, we used BIO and evaluated the effect of upregulation of β-catenin on ALDH^HIGH^ cells. The addition of 5 μM BIO under monoculture conditions led to relocation of β-catenin to the nucleus, a molecular signal of β-catenin activation (Figure 4A). Moreover, there was an increase in media fluorescence when we evaluated the total and active β-catenin using flow cytometry (Supplementary Figures 2A–D). The increases in the transcriptional activity mediated by β-catenin was measured by a pTOP/FOP reporter system in cells treated with BIO, in a dose-dependent manner, confirming that inhibition of GSK3 activated β-catenin (Figure 4B). Nevertheless, the main goal was to know whether β-catenin activation had an effect on ALDH^HIGH^ cells. Remarkably, GSK3 inhibition upregulates ALDH protein levels (Figures 4C,D). In addition to the ALDH protein increases, GSK3 inhibition also elevated the percentage of ALDH^HIGH^ cells, indicating that GSK3 acts upstream of ALDH regulation (Figures 4E,F).

To discern whether the effects on ALDH upon GSK3 inhibition are mediated by β-catenin-related transcriptional activity, we employed a siRNA against β-catenin in addition to BIO treatment. We measured β-catenin-dependent transcription through cells transfected with reporter plasmids pTOP/FOP and observed that inhibition of GSK3 improved the transcriptional activity. In contrast, TCF/LEF transcriptional activity was reduced upon β-catenin knockdown (Figure 4G). To corroborate this effect, we observed that protein levels of ALDH increased upon GSK3 inhibition, and were notably reduced by β-catenin suppression (Figures 4H,J). Impaired activation of β-catenin by siRNA was monitored detecting a non-phosphorylated form of β-catenin that prevents its degradation and favors its transcriptional activity, hereafter referred to as active β-catenin. The results showed that siRNA decreased active β-catenin, demonstrating that, in fact, the decrease of ALDH levels is due to β-catenin, which in turn is negatively regulated by GSK3 (Figures 4H,I). Additionally, we wanted to know if this premise was corroborated in spheres cultures, thus we pre-treated cells during 48 h with 5 μM BIO and then performed the sphere formation assay with the purpose of measuring the percentage of ALDH^HIGH^ cells. In this manner, we show an enrichment of this cellular subpopluation, similarly to monolayer (Supplementary Figures 3A,B).

### ALDH^HIGH^ Cellular Subpopulation Possesses Elements Associated With β-Catenin Pathway Activation

**Figure 5:**
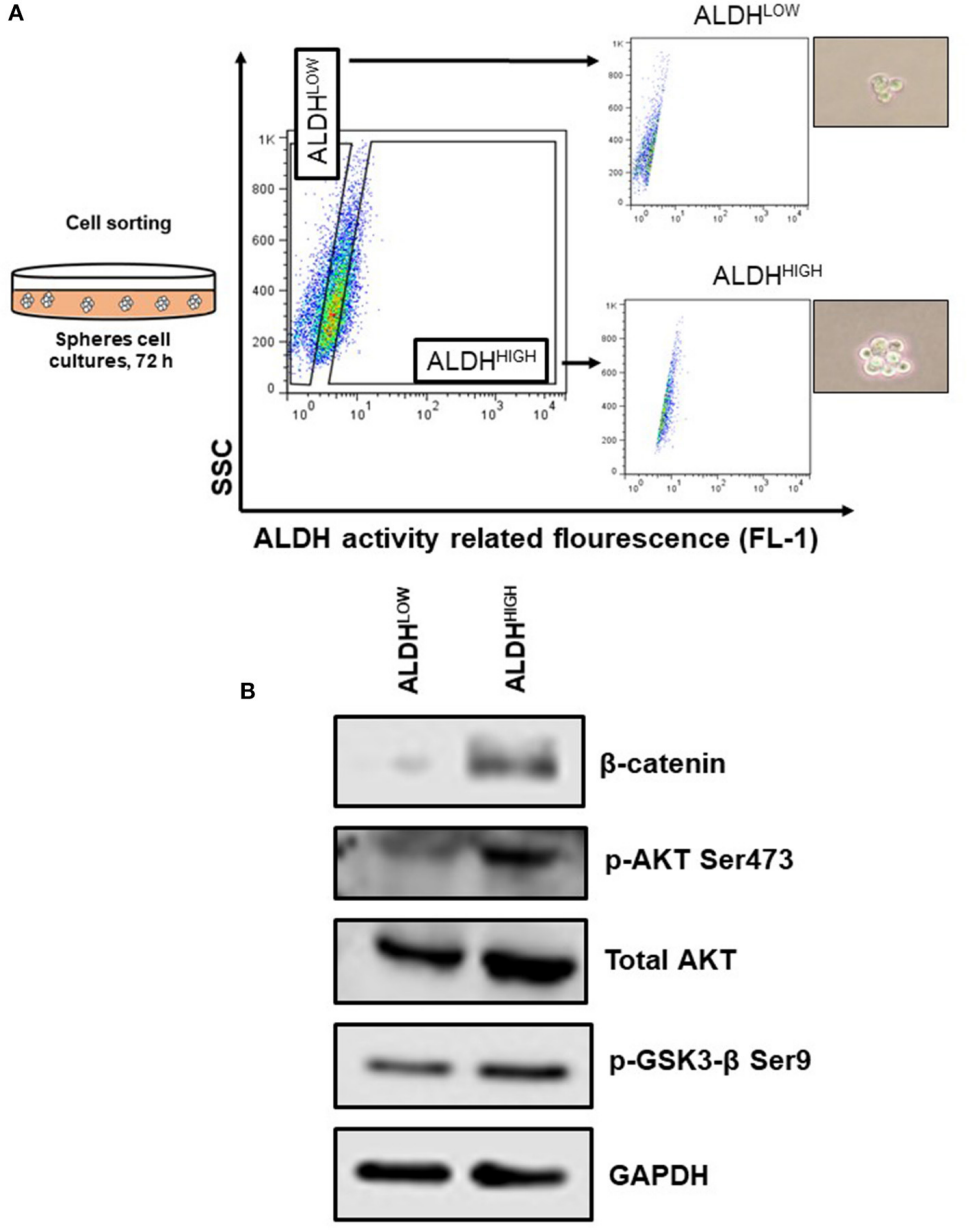
The ALDH^HIGH^ subpopulation from spheres model shown elements associated to the activation of β-catenin. **(A)** Schematic model of cell sorting to ALDH^HIGH^ and ALDH^LOW^ subpopulations from spheres grown 72 h. **(B)** Western Blot analysis of β-catenin, p-GSK3-β Ser9, and p-AKT Ser473 and total AKT from sorted ALDH subpopulations.

Once it was established that ALDH expression and ALDH^HIGH^ cells are regulated by β-catenin, which in turn remains inactive due to the action of GSK3 in SiHa cells, we decide to determine the possible upstream components of this axis, given previous results showing a low percentage of Fzd. Particularly, to address the mechanism favoring Fzd-independent β-catenin activation in the ALDH^HIGH^ subpopulation under sphere formation conditions, ALDH^HIGH^ cells and ALDH^LOW^ cells were isolated by cell sorting from spheres cultures (Figure 5A).

The analysis of ALDH^HIGH^ cells from spheres showed that β-catenin is enriched in this subpopulation, confirming our previous results that sustain β-catenin is important to regulate ALDH protein levels and ALDH^HIGH^ cell percentages. Furthermore, GSK3-β can be inactivated by phosphorylation at specific sites such as Ser9, a post-translational modification that was observed in ALDH^HIGH^ cells, suggesting that the enrichment of β-catenin may be, at least in part, due to the inactivation of GSK3-β in this type of cells (Figure 5B). Noteworthy, phosphorylation at Ser9 in GSK3-β (inactive form) can be attributed to Wnt-independent mechanisms, such as growth factor signals initiated by EGF or FGF (19). One of the negative regulators of GSK3-β is AKT, which phosphorylates Ser9. In accordance with these data, we found that, not only were total AKT levels higher in ALDH^HIGH^ cells, but also the phosphorylated form of AKT at Ser473 (active form) was detected, explaining the inactivation of GSK3-β in our system (Figure 5B).

### β-Catenin and ALDH Are Regulated by AKT

**Figure 6:**
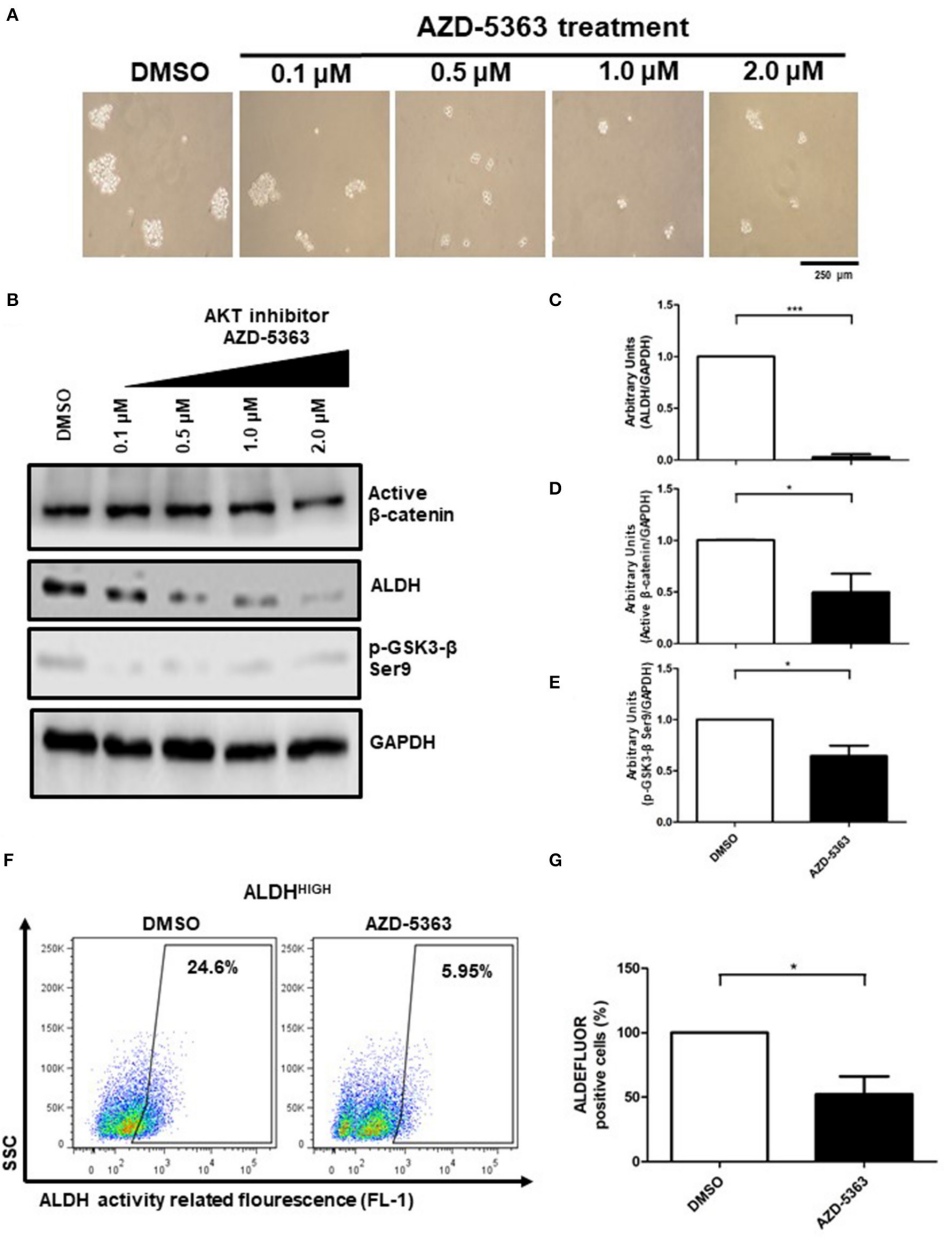
The AKT activity is present in upstream β-catenin/ALDH axis. **(A)** Representative image of spheres size upon 0.1–2.0 μM AZD-5363 treatment. **(B–E)** Western Blot analysis of active β-catenin, p-GSK3-β Ser9, and ALDH protein from spheres treated with 0.1–2.0 μM AZD-5363, the bar graphs shown the comparation of control (DMSO) vs. 2.0 μM AZD-5363 treatment from 3 independent experiments (Means ± S.E.M.), *p < 0.05,***p < 0.001. **(F)** Density plots showing the percentage of ALDH^HIGH^ cells in spheres treated with control (DMSO) or 2.0 μM AZD-5363 treatment for 72 h. **(G)** The bar graph shown the percentage of ALDEFLUOR positive cells in both conditions, DMSO and AZD-5363 treatment, considering 3 independent experiments (Means ± S.E.M.), *p < 0.05.

As previous findings suggested, activation of AKT can explain the enrichment of ALDH^HIGH^ cells in spheres, thus we decided to determine whether AKT inhibition affect β-catenin and ALDH activities. To elucidate this, we inhibited AKT using AZD-5363 inhibitor. First, we observed a decrease in the size of spheres in a dose-dependent manner (Figure 6A). Notably, the AKT inhibitor reduced the active form of β-catenin as well as the inactive form of GSK3-β, both detected in ALDH^HIGH^ cells. Furthermore, ALDH protein levels were reduced upon AZD-5363 treatment, suggesting that AKT activity is upstream of these components (Figures 6B–E). Finally, flow cytometry analysis allowed us to corroborate that AKT inhibition impacts negatively on the percentage of ALDH^HIGH^ cells, reducing this subpopulation in spheres (Figures 6F,G).

## Discussion

ALDH is an enzyme superfamily encoded by 19 genes and 3 pseudogenes, described in the human genome. Moreover, the widespread tissue distribution of ALDH enforces the importance of this enzymatic activity on different cellular processes. Although there are several isoforms and levels depending on the cellular context, the cells with the highest ALDH activity partially explain drug resistance in cancer (1, 20). Based on interaction with the ALDH1A1 promoter region, it has been demonstrated that it is positively regulated by transcriptional activity of β-catenin. Furthermore, putative TCF/LEF binding sites on the ALDH1B1 promoter have been identified. The inhibition of ALDH enzymatic activity results in a decrease of tumorigenic capacities as well as properties associated with Cancer Stem Cells (21). We observed an association with low clinical survival in the case of ALDH1B1 in CC samples, but we must consider the participation of other ALDH isoforms to improve the ALDEFLUOR positivity, including ALDH3A1, ALDH7A1, ALDH2, among others (22). Basically, in CC as well as different types of cancer, several reports sustain there is increased presence of ALDH1A1 with respect to normal tissues. Moreover, using tridimensional cell cultures we have been able to characterize the expression of ALDH1A1 and ALDH1A3 and have associated their expression with stemness properties (Manuscript in preparation, Toledo-Guzman M).

The over-activation of the Wnt pathway, the main signaling mechanism in which β-catenin participates, is a characteristic described in many types of cancer, mainly colon, where inactivating mutations in negative regulators such as APC (Adenomatous Polyposis Coli) or β-catenin itself have been detected in most cases (8, 9, 12). However, tissues and cell lines obtained from CC, have alterations in the canonical Wnt pathway, and such alterations are related to the inhibition of typical antagonists such as Wnt Inhibitory Factor 1 (WIF1), DKK3, Soluble Frizzled-Related Proteins (SFRP) or APC (13, 23, 24). In this work, we observed that β-catenin is mainly localized in membrane and cytoplasm, as well as low β-catenin-dependent transcriptional activity (data not shown), which indicates that over-activation of canonical Wnt pathway is not necessary in these cancer cells, as noted occasionally in biopsies of patients with CC. Nevertheless, the presence of nuclear β-catenin is related with poor clinical prognosis in CC, suggesting that its transcriptional activity is relevant to malignant progression in this type of cancer (11, 25). For example, the Fujimori group has proposed that β-catenin displays an abnormal distribution pattern in 65% of tissues analyzed, being a clinical factor associated with progression of cervical adenocarcinoma to an invasive phenotype (14).

The activation of β-catenin, in our model, through GSK3 inhibition, lead to higher levels of ALDH protein and ALDH^HIGH^ cell proportion. It is important to emphasize that upregulation of ALDH levels has implications in cellular signaling independently of its enzymatic activity. For instance, ALDH has been demonstrated to stabilize GLI2, a transcription factor involved in Sonic-Hedgehog signaling pathway, through a mechanism where enzymatic activity is dispensable, impacting cellular functions such as proliferation and migration (26).

Notably, the activation of β-catenin also increased the percentage of cells with high ALDH activity (27). The ALDH^HIGH^ cells have been associated with higher tumorigenic capacity, particularly in CC cell lines, suggesting that ALDH mediate the relationship between nuclear β-catenin in samples of patients with CC and poor clinical prognosis (27). Moreover, ALDH has been related with lower sensitivity to pharmacological treatments like cisplatin, highlighting its importance in explaining some pharmacological resistance (3).

Although ALDH^HIGH^ cells have become relevant due to their properties, several studies have described that the percentage of these cells is low in standard adherent culture conditions. For this reason, we enriched this cellular subpopulation in a sphere formation assay, finding that this enrichment is dependent on the presence of β-catenin. Despite this relevance, we do not exclude the possible role of other regulatory elements such as SOX9 or C/EBP beta that can also regulate ALDH expression (28, 29). Similarly, we and other groups have reported that the sphere model reflects the presence of other stemness cellular markers besides ALDH^HIGH^ subpopulation. These reports highlight the regulatory role of β-catenin in establishing a malignant phenotype (4, 15).

There are different stimuli regulating β-catenin activity, the best known being Wnt signaling. The Wnt ligands, through their effect on Fzd receptor and LRP coreceptor, promote the dissociation of the protein complex that normally favors β-catenin degradation (8, 30). Nevertheless, in our model we observed low percentage of Fzd positive cells, and no association of this receptor with overall survival in patients with CC, highlighting the existence of other mechanisms explaining the activation of the β-catenin/ALDH axis.

**Figure 7:**
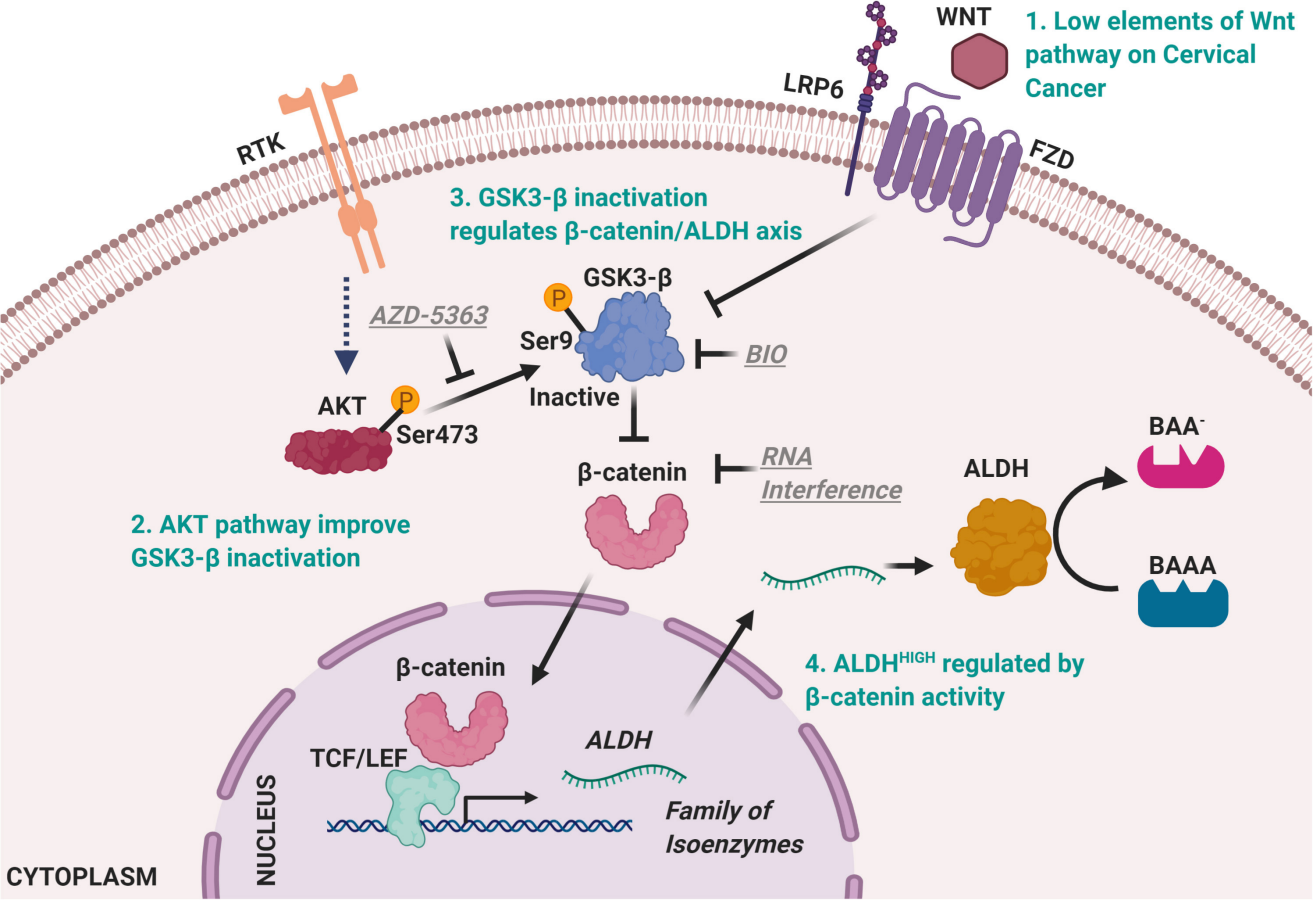
Model of ALDH^HIGH^ cells regulatory mechanisms in cervical cancer cells. ALDH^HIIGH^ cells are present in low percentage in monolayer culture. However, extracellular stimuli perform the enrichment of ALDH^HIGH^ cells during spheres formation, possibly through RTK like receptors able to promote PI3K/AKT pathway, given the low percentage of Fzd^+^ cells and that blocking effect of AZD-5363 over AKT maintained ALDH^HIGH^ cells at low percentage. Thereupon, AKT kinase leads GSK3-β phosphorylation at Ser9 residue to inactivate it. The inactive form of GSK3-β triggers β-catenin stability and once β-catenin levels are elevated into the cell promotes β-catenin translocation to the nucleus to mediate target genes expression including ALDH genes, according to previous reports (13). The ALDH levels elicit a major percent of cells characterized by high activity of ALDH. Figures were created with biorender.com.

Previous evidence has described that β-catenin can also be regulated by growth factors, through the PI3K/AKT pathway. Thus, we explored elements associated with the activation of this signaling pathway in the ALDH^HIGH^ subpopulation. Interestingly, in this subpopulation not only was β-catenin present, but also p-AKT Ser473 (active form). In the regulation of PI3K/AKT over β-catenin, different posttranslational modifications have been associated with the activity of β-catenin. One of these is the inactivation of GSK-3β through phosphorylation at Ser9, being independent of Wnt ligands (19, 31). Consistently, in the ALDH^HIGH^ subpopulation obtained by cell sorting, we detected p-GSK-3β Ser9, pointing to its regulation of the β-catenin/ALDH axis in this cellular context as being regulated by receptor tyrosine kinases (RTK). Taking together, these results allow us to propose the molecular axis regulating ALDH^HIGH^ population in the sphere model (Figure 7). These results are in accordance with reports in nasopharyngeal cancer and propose a less described mechanism for the regulation of the β-catenin/ALDH axis in CC (32). This evidence is reinforced by recent clinical data where cervical intra-epithelial neoplasia and squamous cell carcinoma show higher levels of p-GSK-3β Ser9 with respect to normal tissues (33).

Likewise, the activation of RTKs, such as Epidermal Growth Factor Receptor (EGFR), has been associated with ERK2 and β-catenin activity, which through CK2 act over α-catenin improving its phosphorylation. The consequence of this mechanism is the disruption of β-catenin and α-catenin interaction and increased β-catenin transcriptional activity over TCF/LEF (34). Another example of Wnt-independent mechanisms over β-catenin is the activation of PI3K/AKT, which can act at several points over β-catenin through loss of cellular polarity. AKT acts either to inactivate GSK-3β or directly over β-catenin through phosphorylation at ser552, improving the dissociation of β-catenin from adherent junctions and thus, favoring its function as transcriptional coactivator (35).

Our results reveal the existence of mechanisms with oncogenic progression regulated by PI3K/AKT observed in the ALDH^HIGH^ subpopulation, given that pharmacological inhibition of AKT decreased the enrichment of ALDH^HIGH^ cells, as well as the proteins involved in its activity. Additionally, we must consider the elements present in the ALDH^HIGH^ subpopulation regulated downstream of the PI3K/AKT axis, including mTORC1 activity. It has been reported that pharmacological inhibition of mTORC1 reduces the ALDH^HIGH^ subpopulation. Moreover, targets such as c-Myc and CyclinD1 are regulated directly at the translational level by AKT, so we should not limit the effect of β-catenin to oncogenic capabilities (36, 37). Furthermore, while this work emphasizes the role of PI3K/AKT on presence and activity of ALDH, the possible participation of HPV oncoproteins must not to be excluded, since these have been associated to β-catenin activation and this could directly or indirectly explain ALDH regulation (38, 39).

In summary, our results reveal AKT as a key upstream regulator to explain the up-regulation of β-catenin/ALDH^HIGH^ activity subset in a Fzd-independent mechanism, corroborating the associated clinical data in cervical cancer. Studying mechanisms involved in β-catenin activation, will allow us to elucidate the regulation of properties such as stemness, survival, and low clinical prognosis related to ALDH^HIGH^ activity.

## Data Availability Statement

The datasets presented in this study can be found in online repositories. The names of the repository/repositories and accession number(s) can be found in the article/Supplementary Material.

## Author Contributions

MS-S and EA-O participated in the experimental design and writing of this manuscript. MS-S, EA-O, and MT-G performed the experiments and analyzed the data. AG-C and EO-S reviewed and suggested modifications to the content of this manuscript. AG-C and EO-S provided the financial support. EO-S supervised and approved the final version of the article. All authors contributed to the article and approved the submitted version.

## Conflict of Interest

The authors declare that the research was conducted in the absence of any commercial or financial relationships that could be construed as a potential conflict of interest.

## Supplementary Material

Supplementary Figure 1 Distribution of active β-catenin in nuclear and cytoplasmic fractions. **(A)** Western Blot of the active form of β-catenin from monolayer (ML) and spheres (SP) cell cultures grown for 3 days. GAPDH and Lamin A/C were used as loading control for cytoplasmic and nuclear fractions. **(B)** The percentage of the active form of β-catenin was calculated normalizing to loading control corresponding to each fraction. Quantification of 4 independent experiments is shown in bar graphs (Means ± S.E.M), ^**^*p* < 0.01.

Supplementary Figure 2 BIO increases the β-catenin levels and β-catenin-dependent transcriptional activity. **(A,B)** Western Blot of β-catenin in SiHa cells growth as monolayer culture with BIO treatment at the concentrations indicated during 48 h. GAPDH was used as loading control. Densitometry quantification is shown in the bar graph (Means ± S.E.M), ^*^*p* < 0.05. **(C,D)** Histograms of β-catenin and non-phosphorylated β-catenin in SiHa cells exposed to DMSO (red line) or 5 μM of BIO (blue line) during 48 h determined by flow cytometry. The media fluorescence was normalized to control (DMSO) and the data from at least 3 independent experiments are shown in bar graph, (Means ± S.E.M.), ^*^*p* < 0.05, ^***^*p* < 0.001.

Supplementary Figure 3 GSK3-β inhibition upregulates the percentage of ALDH^HIGH^ cells in spheres. **(A,B)** Density plots showing the percentage of ALDH^HIGH^ cells obtained of spheres cultures from SiHa cells pretreated with BIO. SiHa cells were growth in monolayer and treated with DMSO (vehicle) or 5 μM of BIO. At 48 h of treatment, the cells were seed and growth under spheres cultures for an additional 72 h. The enzymatic activity of ALDH was measured by ALDEFLUOR kit. The percentages were normalized with DEAB (internal control) and the data from 6 independent experiments are shown in bar graph (Means ± S.E.M.). ^*^*p* < 0.05.
