# Supplementary figures and images for: ALDHHIGH Population Is Regulated by the AKT/β-Catenin Pathway in a Cervical Cancer Model
Source: Front Oncol. 2020 Jul 17;10:1039. doi: 10.3389/fonc.2020.01039 (PMC7379485; doi:10.3389/fonc.2020.01039)

Supplementary Figure 1

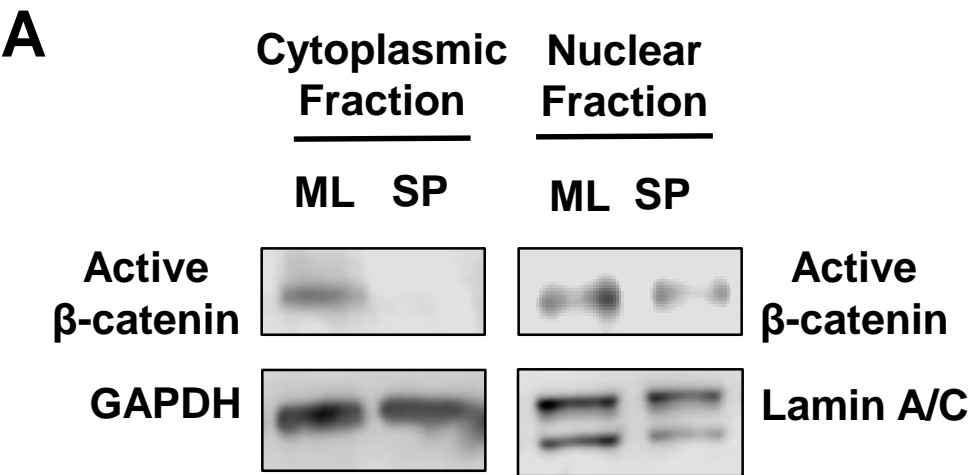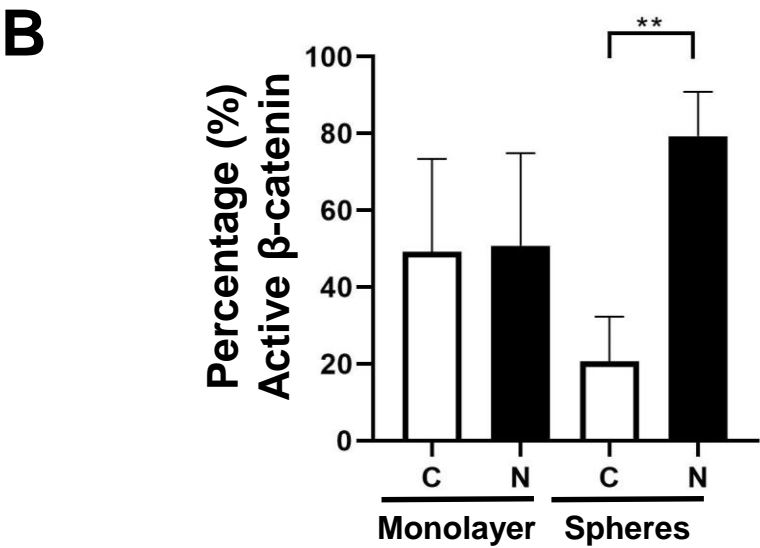

Supplement: Supplementary Figure 1 — Distribution of active β-catenin in nuclear and cytoplasmic fractions. (A) Western Blot of the active form of β-catenin from monolayer (ML) and spheres (SP) cell cultures grown for 3 days. GAPDH and Lamin A/C were used as loading control for cytoplasmic and nuclear fractions. (B) The percentage of the active form of β-catenin was calculated normalizing to loading control corresponding to each fraction. Quantification of 4 independent experiments is shown in bar graphs (Means ± S.E.M), **p < 0.01. [file Image_1.pdf]

## Supplementary Figure 2

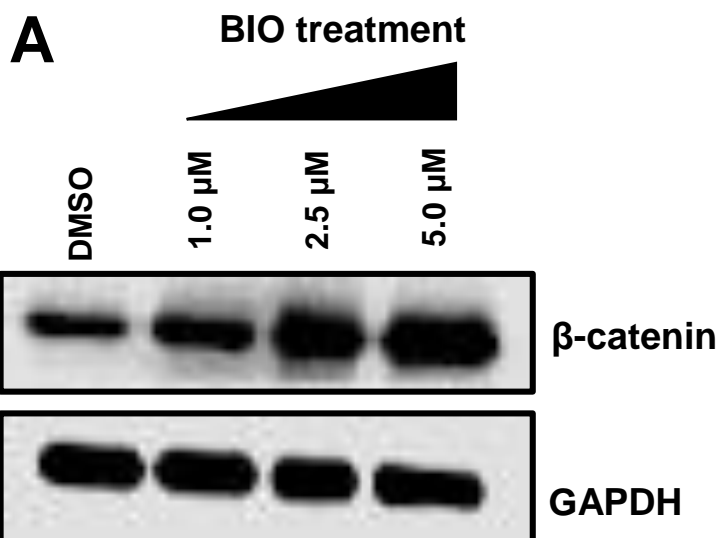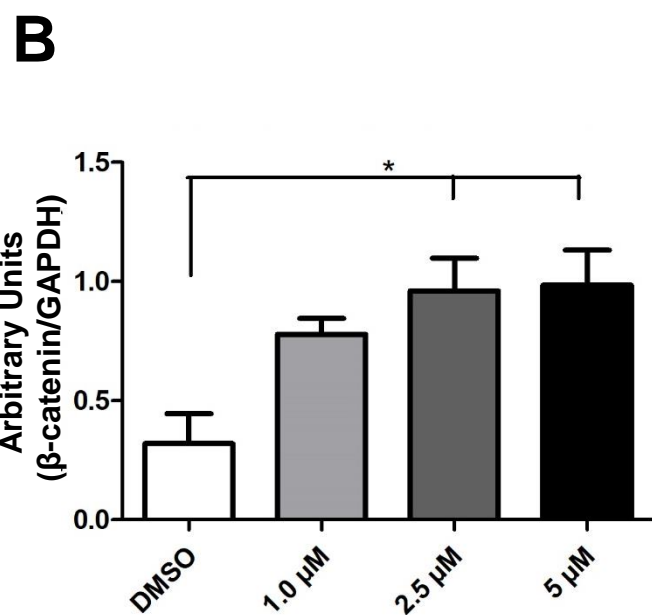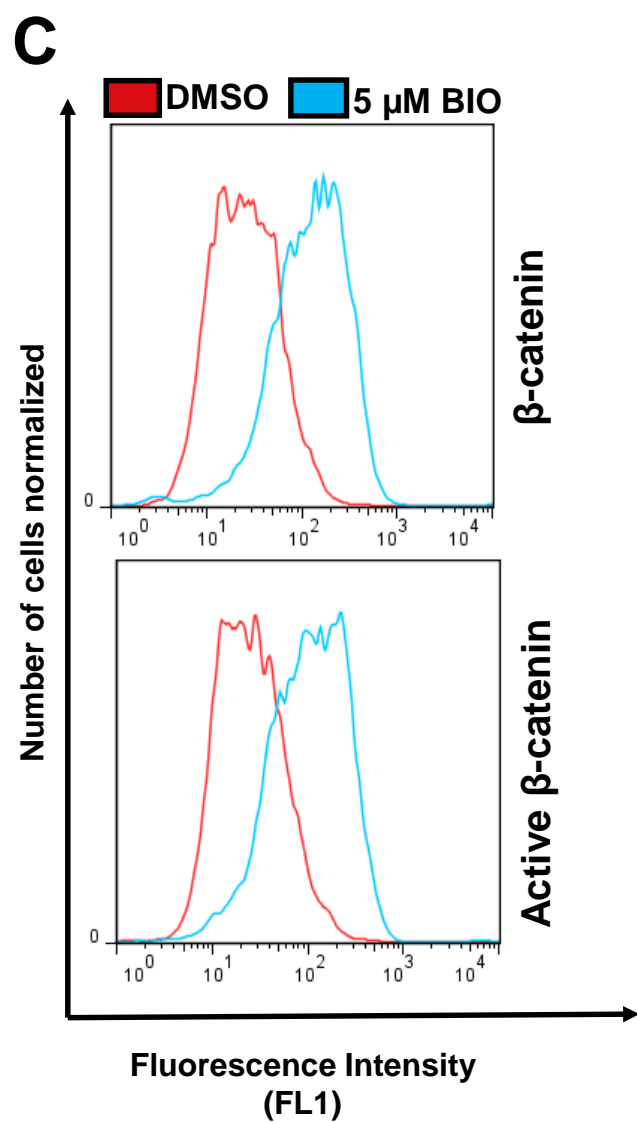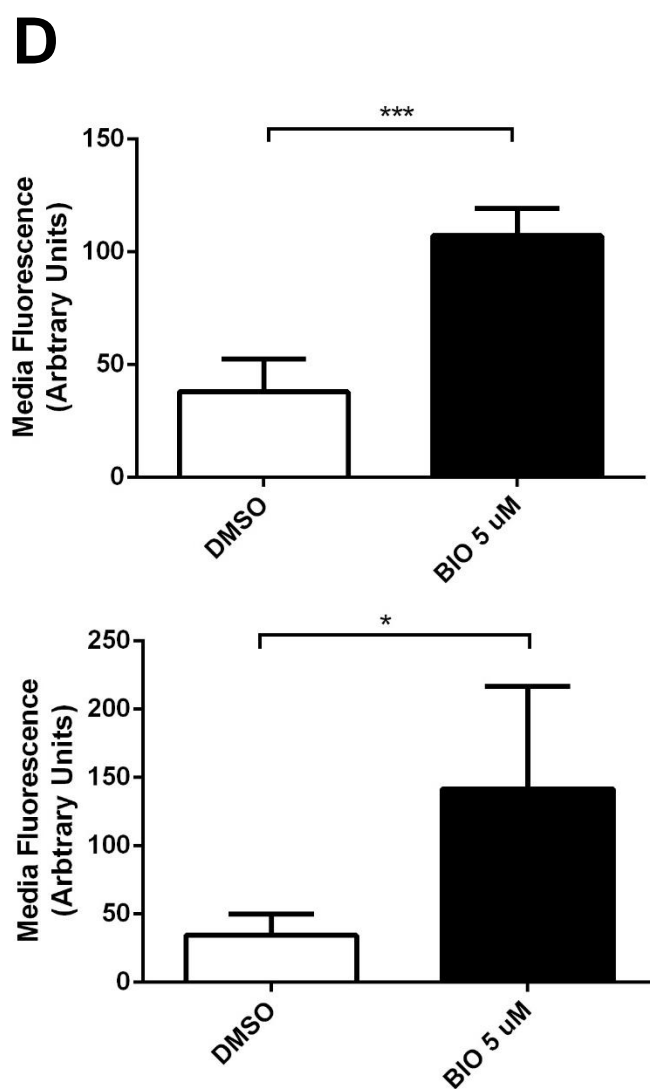

Supplement: Supplementary Figure 2 — BIO increases the β-catenin levels and β-catenin-dependent transcriptional activity. (A,B) Western Blot of β-catenin in SiHa cells growth as monolayer culture with BIO treatment at the concentrations indicated during 48 h. GAPDH was used as loading control. Densitometry quantification is shown in the bar graph (Means ± S.E.M), *p < 0.05. (C,D) Histograms of β-catenin and non-phosphorylated β-catenin in SiHa cells exposed to DMSO (red line) or 5 μM of BIO (blue line) during 48 h determined by flow cytometry. The media fluorescence was normalized to control (DMSO) and the data from at least 3 independent experiments are shown in bar graph, (Means ± S.E.M.), *p < 0.05, ***p < 0.001. [file Image_2.pdf]

Supplementary Figure 3

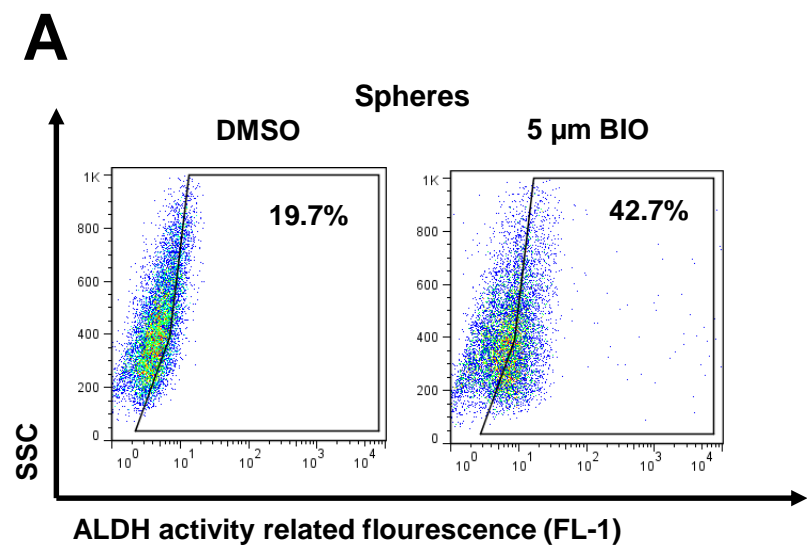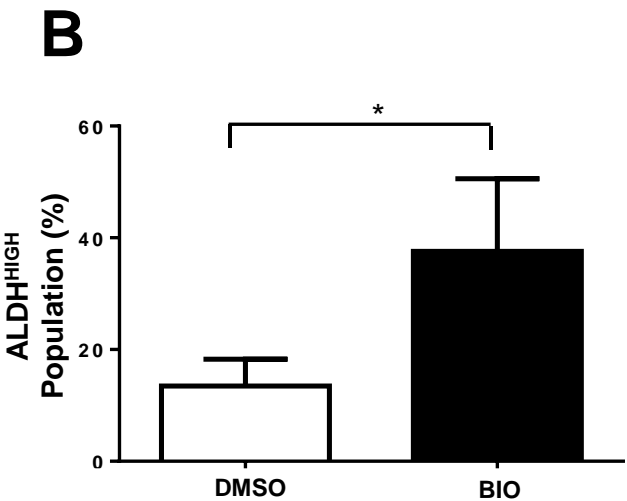

Supplement: Supplementary Figure 3 — GSK3-β inhibition upregulates the percentage of ALDHHIGH cells in spheres. (A,B) Density plots showing the percentage of ALDHHIGH cells obtained of spheres cultures from SiHa cells pretreated with BIO. SiHa cells were growth in monolayer and treated with DMSO (vehicle) or 5 μM of BIO. At 48 h of treatment, the cells were seed and growth under spheres cultures for an additional 72 h. The enzymatic activity of ALDH was measured by ALDEFLUOR kit. The percentages were normalized with DEAB (internal control) and the data from 6 independent experiments are shown in bar graph (Means ± S.E.M.). *p < 0.05. [file Image_3.pdf]
